# What is missing? Addressing the complex issues surrounding sexual and reproductive health in the circumpolar north

**DOI:** 10.3402/ijch.v75.34287

**Published:** 2016-12-09

**Authors:** Cornelia Jessen, Jessica Leston, Brenna Simons, Elizabeth Rink

**Affiliations:** 1 Division of Community Health, Alaska Native Tribal Health Consortium, Anchorage, AK, USA; 2 Northwest Portland Area Indian Health Board, Tribal Epidemiology Center, Portland, OR, USA; 3 The Center for American Indian and Rural Health Equity, Montana State University, Bozeman, MT, USA

Across the globe, sexual and reproductive health (SRH) is a complex issue, with a myriad of moving parts and diverse stakeholders from different knowledge and value systems. In the Arctic, the context of SRH and associated health disparities are composed of psychological, social, cultural, economic, educational, geographic and political factors. There is a dearth of published literature on the basic epidemiology of sexually transmitted infections (STI), and even fewer publications on teen pregnancy and domestic violence in the Arctic. But as a community of circumpolar healthcare and public health professionals, we know that impact of SRH goes beyond the published scholarship available on epidemiology and surveillance. More publications regarding the existence of these disparities and the ways to address SRH through research and programmes that take into account the unique circumstances found within Arctic communities are needed. Discussions regarding increasing the number of publications that explore the many interconnected pieces of SRH in the circumpolar north began at the 16th International Congress on Circumpolar Health in Oulu, Finland, in June 2015, among members of the new Circumpolar Network for Sexual and Reproductive Health.

The result of these early conversations is this special issue which aims to present current social science research and programme activities that address the complexity of SRH in the Arctic, with particular emphasis on indigenous health. The Call for Papers solicited original work with the following foci:

- Innovative curricula, interventions and educational programmes implemented in arctic environments.
- Improving access to SRH care services in Arctic communities.
- Efforts to address the social, individual, psychological, environmental and cultural variables affecting SRH in the Arctic.
- Research ethics, challenges and lessons learned of addressing sensitive topics such as SRH in arctic environments.
- Social science research and programmatic science partnerships and collaborations on SRH between the circumpolar countries.
- Indigenous perspectives on SRH opportunities and promotion efforts.

The special issue received numerous submissions on SRH. Themes that emerged from the final manuscripts include sexual violence, teen pregnancy, STI, breastfeeding and maternal health, representing almost all topical areas described in the Call for Papers. Collectively, the articles speak to the diversity of individual, community and systems approaches needed to effectively influence SRH within the circumpolar context and establish evidence that provides recommendations for community-based participatory research (CBPR), inclusion of indigenous ways of knowing and methodologies, and a need for culturally responsive programmes that are respectful of indigenous self-determination, place, people and culture.

For instance, CBPR was applied to gain insight into youth perspectives on sexual health (1) as well as perceived barriers experienced by youth in accessing care and resources (2), and Inuit women's perspectives on HIV and STI prevention and sexual health promotion (3). The authors also reflected on programme successes and challenges which strongly support the argument that location and individuality of community directly impact the success of CBPR (4), including the recognition that diversity and specific needs exist even within small, isolated Arctic communities (5). Systematic literature reviews revealed a need for more culturally relevant health indicators (6) and community-specific epidemiology (7). Finally, programme evaluations included in this special issue strengthen the concept that each community can be unique despite similar health disparities identified through regional epidemiology. As a result, programmes must be adapted to fit the local context by measures such as creating local provider awareness (8), tailoring clinical services (9) and individualizing social services to address child and sexual abuse (10).

It is our hope that the papers included in this special issue provide examples of what is required of researchers, programme staff and communities in the circumpolar north when addressing SRH within the complexity of local context. Each of the papers in this special issue helps to provide strategies to address the challenges we face as SRH researchers and programme planners and implementers, and encourages us to work in partnership with Arctic communities to define problems and find appropriate solutions. The editors are grateful for the dedicated work of all those who submitted their paper to this special issue and are thankful for each contributor's ability to take nuanced adaptive steps towards alleviating SRH disparities through advancing the science of SRH research and programming in the Arctic.

You can find the International Union for Circumpolar Health Sexual Health Working Group on Facebook and LinkedIn.

This special issue was made possible by the U.S. Indian Health Service HIV Program and the U.S. Department of Health & Human Services, Secretary's Minority AIDS Initiative Fund.

*Cornelia Jessen* Division of Community Health Alaska Native Tribal Health Consortium Anchorage, AK, USA *Jessica Leston* Northwest Portland Area Indian Health Board Tribal Epidemiology Center Portland, OR, USA *Brenna Simons* Division of Community Health Alaska Native Tribal Health Consortium Anchorage, AK, USA *Elizabeth Rink* The Center for American Indian and Rural Health Equity Montana State University Bozeman, MT, USA
